# Supplementary material for: Practical GHz single-cavity all-fiber dual-comb laser for high-speed spectroscopy
Source: Light Sci Appl. 2025 Mar 24;14:133. doi: 10.1038/s41377-025-01811-3 (PMC11930957; doi:10.1038/s41377-025-01811-3)
Supplement: Supplementary file 1 — Supplementary information [file 41377_2025_1811_MOESM1_ESM.docx]

**Supplementary Information for:**

**Practical GHz single-cavity all-fiber dual-comb laser for high-speed spectroscopy**

Lin Ling^1,†^, Wei Lin^1,†^, Zhaoheng Liang^1,†^, Minjie Pan^1^, Chiyi Wei^1^, Xuewen Chen^1^, Yang Yang^1^, Zhijin Xiong^1^, Yuankai Guo^1^, Xiaoming Wei^1,2*^, and Zhongmin Yang^1,2,3*^

^1^School of Physics and Optoelectronics, South China University of Technology, Guangzhou 510640, China.

^2^School of Materials Science and Engineering; Guangdong Engineering Technology Research and Development Center of Special Optical Fiber Materials and Devices; Guangdong Provincial Key Laboratory of Fiber Laser Materials and Applied Techniques, South China University of Technology, Guangzhou 510640, China.

^3^Research Institute of Future Technology, South China Normal University, Guangzhou 510006, China.

^†^These authors equally contribute to this work.

^*^Correspondence should be addressed to X.M.W. (xmwei@scut.edu.cn) or Z.M.Y. ([yangzm@scut.edu](mailto:yangzm@scut.edu).cn).

**Supplementary Note 1:** **Experiment setup of the dual-wavelength mode-locked fiber laser (MLFL)**

**Figure S1** illustrates the experimental setup of the dual-wavelength MLFL. The laser cavity consists of an 8.5-cm heavily Yb-doped few-mode gain fiber (FMGF), a 0.8-cm single-mode fiber (SMF), a semiconductor saturable absorber mirror (SESAM), and a fiber-based dielectric film (DF). The FMGF is fusion-spliced together with the SMF, while the unspliced end facet of the FMGF is butt-coupled to the DF. The opposite unspliced end facet of the SMF is attached to the SESAM. The DF with multilayer stack is coated onto the fiber end facet in a ceramic ferrule and the pigtail is fusion-spliced to the common port of a 976/1064 nm wavelength-division multiplexer (WDM). The DF has a reflectivity of 85% at the signal wavelength and a transmittance of 90% at the pump wavelength. The SESAM (Batop GmbH, SAM-1040-8-1ps-1.0-0) has a modulation depth of 5%, a recovery time of 1 ps, and a saturated fluence of 40 μJ·cm^-2^. The single-mode laser diode (SM-LD) centered at 976 nm with a maximum power of 460 mW serves as the pump source, which is coupled into the cavity through the WDM. A 90:10 optical coupler (OC) is utilized to split the MLFL signal into two parts, and the 10% part is used for monitoring. A fiber isolator (ISO) protects the laser cavity from backward reflection. The dual-wavelength optical spectrum is separated by a filter wavelength-division multiplexer (FWDM), with the 1062-nm part transmitted while the 1056-nm part reflected. The whole laser cavity is temperature-controlled at 20 ℃ via a thermo-electric cooler (TEC) for stable long-term mode-locking state.

**Fig. S1** **Experi****ment setup of the dual-wavelength mode-locked fiber laser (MLFL).** SESAM, semiconductor saturable absorber mirror; SMF, single-mode fiber; FMGF, few-mode gain fiber; DF, dielectric film; WDM, wavelength-division multiplexer; SM-LD, single-mode laser diode; OC, optical coupler; ISO, isolator; FWDM, filter wavelength-division multiplexer; TEC, thermo-electric cooler.

**Supplementary Note 2:** **Multimode interference (MMI)-mediated spectral filtering effect in the ultrashort fiber cavity**

In this work, the Yb-doped FMGF is employed as the gain medium of the laser cavity. It has a core diameter $d$ of 6.4 μm and a numerical aperture $NA$ of 0.148. Accordingly, as operating at a center wavelength $\lambda$ of 1059 nm, the relevant normalized frequency $V$ that is calculated as

$$\begin{aligned} V=\frac{\pi d}{\lambda}\cdot NA=2.809\#\left( S1 \right) \end{aligned}$$

which exceeds the critical condition of fundamental mode guiding (i.e., $V_{c}$ = 2.405). Thus, this FMGF operates in the few-mode regime, and the modal analysis reveals the presence of both fundamental mode (i.e., LP_01_) and high-order mode (HOM, i.e., LP_11_), as shown in **Fig. S2a**. Their group velocity difference as a function of the operating wavelength is given in **Fig. S2b**. As the laser oscillates in the linear cavity, its single round-trip pass can be considered as an SMF-FMGF-SMF structure, as shown in the inset of **Fig. S2c**. The intermodal group velocity difference between the fundamental mode and HOM leads to temporal walk-off, and mutual coupling results in MMI.

To analyze the MMI-mediated spectral filtering effect in the fiber cavity, assuming only first-order dispersion is taken into account, we have

$$\begin{aligned} 2L\delta\beta_{1}\Delta\omega=2\pi\#\left( S2 \right) \end{aligned}$$

where $\delta\beta_{1}$ is the group velocity difference between the transverse modes, $\Delta\omega$ is the frequency spacing of the interference fringes, and *L* is the length of the FMGF (i.e., 8.5 cm). The corresponding wavelength spacing $\Delta\lambda$ can be calculated by

$$\begin{aligned} \Delta\lambda=\left| \frac{\lambda^{2}}{2Lc\delta\beta_{1}} \right|\#\left( S3 \right) \end{aligned}$$

as provided in **Fig. S2c**.

**Fig. S2 Multimode interference (MMI)-mediated spectral filtering effect in the ultrashort fiber cavity. a** Intensity profiles of the transverse modes LP_01_ and LP_11_. **b** Group velocity difference between fundamental mode (LP_01_) and high-order mode (HOM, primarily LP_11_). The inset table provides the key parameters of the FMGF. **c** Wavelength spacing $\Delta\lambda$ of the MMI-mediated spectral filter. Inset shows the SMF-FMGF-SMF structure.

**Supplementary Note 3: Experimental control of mode proportions in the FMGF**

The mode proportions in the FMGF are achieved by adjusting the coupling condition of the light as it transmits from the external-cavity SMF (connected to the WDM) to the intracavity FMGF. This is implemented by precisely rotating the ferrules at the coupling interface between the SMF and FMGF (**Fig. S3a**), which can influence the transverse spatial-hole burning effect by pump coupling and result in different mode proportions in the FMGF^1^.

To further validate this, we use a single-pass configuration with SMF and FMGF (inset of **Fig. S3b**) and conduct the mode decomposition of the beam profiles based on a non-iterative strategy^2^ for different coupling conditions. By virtue of a pseudoinverse matrix, the complex coefficient $C_{k}$ involving the amplitude and relative phase of the spatial mode $k$, namely,

$$I^{\left( m \right)}=\sum_{k} \sum_{j} C_{k}C_{j}^{*}\Psi_{k}^{\left( m \right)}\Psi_{j}^{\left( m \right)}, \text{ }m=1,\text{ ..}, M^{2}\text{ } (S4)$$

can be retrieved. In the equation, the measured intensity profile is manifested as an image with $M\times M$ pixels, and $\Psi_{k}$ characterizes the eigenmode of the FMGF. According to this scheme, as shown in **Fig. S3b**, the corresponding proportions of the HOM (i.e., LP_11_ in our work) are 2.39%, 8.48%, and 14.07%, respectively, thus confirming the experimental control of the HOM’s proportion in the FMGF by varying the coupling condition.

In addition, **Fig. S3c** shows the real (top panel) and reconstructed (bottom panel) beam profiles under three different coupling conditions. To showcase the accuracy of the mode decomposition algorithm, the Pearson correlation coefficient (PCC) in terms of

$$PCC=\left| \frac{\iint\left( I-\bar{I} \right)\left( I^{recov}-\bar{I^{recov}} \right)dxdy}{\sqrt{\iint\left( I-\bar{I} \right)^{2}dxdy\iint\left( I^{recov}-\bar{I^{recov}} \right)^{2}dxdy}} \right|\text{ } (S5)$$

is also calculated. The related values exceed 99% in all three cases.

**Fig. S3 Experimental control of mode proportions in the FMGF. a** Configuration of mode coupling adjustment. It is achieved by manually rotating the ferrules at the coupling interface between the SMF and FMGF, such that it can change the coupling position of the fundamental-mode light field as it enters the laser cavity. **b** Mode proportions of the fundamental mode (LP_01_, orange bar) and HOM (LP_11_, green bar) for three different coupling conditions. Inset shows the single-pass configuration used for experimental verification. CCD, charge-coupled device. **c** Real (top) and reconstructed (bottom) beam profiles corresponding to three states in (**b**), from left to right. PCC, Pearson correlation coefficient.

**Supplementary Note 4:** **Transmission curve of the MMI-mediated spectral filter**

To characterize the MMI-mediated spectral filtering effect, we measure its transmission curve using a homemade amplified spontaneous emission (ASE) source. As shown in the top configuration of **Fig. S4a**, we first record an optical spectrum of the ASE source as the reference (purple curve of **Fig. S4b**), which is reflected by a DF reflector through a circulator (CIR). Then, the MMI-mediated spectral filter (i.e., the SMF-FMGF-SMF structure) is inserted, as shown in the bottom configuration of **Fig. S4a**, and the optical spectrum imprinted by the transmission of the MMI-mediated spectral filter is recorded, i.e., the orange curve of **Fig. S4b**. By comparing with the reference, the transmission curve of the MMI-mediated spectral filter is obtained and plotted in **Fig. S4c**. The periodic transmission peaks with a wavelength spacing of 6.28 nm are clearly illustrated, which is close to the calculated filter spacing at an operating wavelength of 1059 nm (i.e., 6.31 nm, **Fig. S4c**).

**Fig. S4 Transmission curve of the MMI-mediated spectral filter.** **a** Experimental setups of measuring the transmission curve. ASE, amplified spontaneous emission; CIR, circulator; OSA, optical spectrum analyzer. **b** Optical spectra of an ASE source without (purple) and with (orange) passing through the MMI-mediated spectral filter. **c** Transmission curve of the MMI-mediated spectral filter.

**Supplementary Note 5: Theoretical study of GHz dual-wavelength MLFL**

**5.1** **Numerical modeling**

In mode-locked lasers with GHz-level fundamental repetition rates, especially with periodic spectral filtering, the intracavity gain strength can barely support the subsistence of dichromatic pulses, and the involved gain competition may finally give rise to single-wavelength mode locking. We explore the modified gain functions in which the mode-locked pulses at two different center wavelengths have gain spectra determined by dynamic rate equations (DREs)^3,4^. Here, it assumes that the gain responds to the pulse at a single center wavelength, rather than a pair of dichromatic pulses, and the pulse-to-pulse interaction only takes place at the SESAM. Thus, the master equations of intracavity elements can be written as

Gain fiber:

$$\begin{aligned} \frac{\partial u\left( z,t \right)}{\partial z}=-i\frac{\beta_{2}}{2}\frac{\partial^{2}u\left( z,t \right)}{\partial t^{2}}+i\gamma\left| u\left( z,t \right) \right|^{2}u\left( z,t \right)+\frac{g_{u}\left( z,\omega\right)}{2}u\left( z,t \right)\#\left( S6a \right) \end{aligned}$$

$$\begin{aligned} \frac{\partial v\left( z,t \right)}{\partial z}=-i\frac{\beta_{2}}{2}\frac{\partial^{2}v\left( z,t \right)}{\partial t^{2}}+i\gamma\left| v\left( z,t \right) \right|^{2}v\left( z,t \right)+\frac{g_{v}\left( z,\omega\right)}{2}v\left( z,t \right)\#\left( S6b \right) \end{aligned}$$

SESAM:

$$\begin{aligned} u\left( L_{f}^{+},t \right)=\mathcal{F}^{-1}\left\{ \mathcal{F}\left[ u\left( L_{f}^{-},t \right) \right]e^{iL\left( \omega\right)}\sqrt{\frac{R_{a}\left( \omega\right)}{\left( 1-q_{a}-q_{0} \right)}} \right\}\sqrt{\left[ 1-q_{a}-q\left( t \right) \right]}e^{i{\alpha\left[ q\left( t \right)-q_{0} \right]}/2} \# \end{aligned}$$

$$\left( S7a \right)$$

$$\begin{aligned} v\left( L_{f}^{+},t \right)=\mathcal{F}^{-1}\left\{ \mathcal{F}\left[ v\left( L_{f}^{-},t \right) \right]e^{iL\left( \omega\right)}\sqrt{\frac{R_{a}\left( \omega\right)}{\left( 1-q_{a}-q_{0} \right)}} \right\}\sqrt{\left[ 1-q_{a}-q\left( t \right) \right]}e^{i{\alpha\left[ q\left( t \right)-q_{0} \right]}/2} \# \end{aligned}$$

$$\left( S7b \right)$$

$$\begin{aligned} L\left( \omega\right)={{\beta_{2a}\omega}^{2}}/{2!}+{{\beta_{3a}\omega}^{3}}/{3!}+{{\beta_{4a}\omega}^{4}}/{4!}+{{\beta_{5a}\omega}^{5}}/{5!}\#\left( S7c \right) \end{aligned}$$

$$\begin{aligned} \frac{dq}{dt}=-\frac{q\left( t \right)-q_{0}}{T_{a}}-\frac{q\left( t \right)}{E_{a}}\left( \left| u\left( L_{f}^{-},t \right) \right|^{2}+\left| v\left( L_{f}^{-},t \right) \right|^{2} \right) \#\left( S7d \right) \end{aligned}$$

where $u$ and $v$ represent the electric fields for the blueshift and redshift spectral components, respectively. To locate their operating wavelengths, we apply pairwise bandpass filters in the form of

$$\mathrm{for}u, T_{u}=exp\left( -\left( \frac{\omega-\omega_{offset}}{\Delta\omega} \right)^{8} \right)$$

$$\mathrm{for}v, T_{v}=exp\left( -\left( \frac{\omega+\omega_{offset}}{\Delta\omega} \right)^{8} \right)$$

where 2$\omega_{offset}$ corresponds to the wavelength spacing of the periodic transmission curve of the laser cavity, i.e., 6.28 nm here.

To account for the gain spectra $g_{u}\left( z,\omega\right)$ and $g_{v}\left( z,\omega\right)$, two sets of standard DREs are utilized,

$$\begin{aligned} \frac{\partial P_{p\left( u/v \right)}\left( z \right)}{\partial z}=\Gamma_{p}\left[ \sigma_{e}\left( \lambda_{p} \right)N_{2\left( u/v \right)}\left( z \right)-\sigma_{a}\left( \lambda_{p} \right)N_{1\left( u/v \right)}\left( z \right) \right]P_{p\left( u/v \right)}\left( z \right)-\alpha P_{p\left( u/v \right)}\left( z \right) \end{aligned}$$

$$(S8a)$$

$$\pm\frac{\partial P_{u/v}^{\pm}\left( z,\lambda_{k} \right)}{\partial z}=\Gamma_{s}\left[ \sigma_{e}\left( \lambda_{k} \right)N_{2\left( u/v \right)}\left( z \right)-\sigma_{a}\left( \lambda_{k} \right)N_{1\left( u/v \right)}\left( z \right) \right]P_{u/v}^{\pm}\left( z,\lambda_{k} \right)$$

$$-\alpha P_{u/v}^{\pm}\left( z,\lambda_{k} \right)+2\sigma_{e}\left( \lambda_{k} \right)N_{2\left( u/v \right)}\left( z \right)\frac{hc^{2}}{\lambda_{k}^{3}}\Delta\lambda(S8b)$$

$$\frac{N_{2\left( u/v \right)}\left( z \right)}{N_{Yb}}=\frac{\frac{\Gamma_{p}\lambda_{p}}{hcA}\sigma_{a}\left( \lambda_{p} \right)P_{p\left( u/v \right)}\left( z \right)+\frac{\Gamma_{s}}{hcA}\sum_{k=1}^{N} \lambda_{k}\sigma_{a}\left( \lambda_{k} \right)\left[ P_{u/v}^{+}\left( z,\lambda_{k} \right)+P_{u/v}^{-}\left( z,\lambda_{k} \right) \right]}{\frac{\Gamma_{p}\lambda_{p}}{hcA}\left[ \sigma_{a}\left( \lambda_{p} \right)+\sigma_{e}\left( \lambda_{p} \right) \right]P_{p\left( u/v \right)}\left( z \right)+\frac{1}{\tau_{G}}+\frac{\Gamma_{s}}{hcA}\sum_{k=1}^{N} \lambda_{k}\left[ \sigma_{a}\left( \lambda_{k} \right)+\sigma_{e}\left( \lambda_{k} \right) \right]\left[ P_{u/v}^{+}\left( z,\lambda_{k} \right)+P_{u/v}^{-}\left( z,\lambda_{k} \right) \right]}$$

$$(S8c)$$

$$\begin{aligned} N_{1\left( u/v \right)}\left( z \right)+N_{2\left( u/v \right)}\left( z \right)=N_{Yb}\#\left( S8d \right) \end{aligned}$$

and the gain function becomes

$$\begin{aligned} g_{u/v}\left( z,\omega\right)=\Gamma_{s}\left[ \sigma_{e}\left( \lambda_{k} \right)N_{2\left( u/v \right)}\left( z \right)-\sigma_{a}\left( \lambda_{k} \right)N_{1\left( u/v \right)}\left( z \right) \right]-\alpha\#\left( S9 \right) \end{aligned}$$

where $P_{p}$ and $P$ represent the $z$-dependent pump power and signal power, respectively. $N_{2}$ and $N_{1}$ are the upper-level and ground populations, respectively. The subscripts $u$ and $v$ indicate the blueshift and redshift spectral components of the dichromatic pulses, and $\pm$ designates forward and backward propagations. Key parameters used in the numerical simulation are concluded in **Table S1**.

**Table S1** Key parameters used in the numerical simulation

| **1. Laser cavity parameters** | **Value** | 3rd dispersion at (*β_3a_, p*s^3^)  4th dispersion at (*β_4a_, p*s^4^) | 2.4×10^-6^  -4.6×10^-8^ |
| --- | --- | --- | --- |
|  |  | 5th dispersion at (*β_5a_, p*s^5^) | 4.4×10^-9^ |
| Fiber length (*L_f_*, cm) | 9.3 | Linewidth enhancement factor (*α*) | 1 |
| Gain fiber dispersion (*β*_2_, fs^2^·mm^-^^1^) | 30 | **3. Dielectric film parameter** | **Value** |
| Gain fiber nonlinearity (*γ*, W^-1^·km^-1^) | 4.5 | Output ratio (*q_l_*) | 0.15 |
| Offset frequency of artificial filter  ($\omega_{offset}$, THz) | 5.3 | **4. Gain characteristics of the gain fiber** | **Value** |
|  |  | Concentration of Yb ions (*N_Yb_*, m^-3^) | 1.5×10^27^ |
| Bandwidth of artificial filter |  | Pump wavelength (*λ_p_*, nm) | 976 |
| ($\Delta\omega$, THz) | 3 | Absorption cross section (*σ_a_*(*λ_p_*), m^2^) | 1.33×10^-24^ |
| **2. SESAM parameters** | **Value** | Emission cross section (*σ_e_*(*λ_p_*), m^2^) | 1.32×10^-24^ |
| Unsaturable loss (*q_a_*) | 0.03 | Reference signal wavelength (*λ_r_,* nm) | 1059 |
| Modulation depth (*q*_0_) | 0.05 | Upper-state lifetime (*τ_G_*, ms) | 1.84 |
| Saturation energy (*E_a_*, pJ) | 5.7 | Core area (*A*, μm^2^) | 28.3 |
| Relaxation time (*T_a_*, ps) | 1 | Overlap factor (*Γ_p_*, *Γ_s_*) | 0.85 |
| 2nd dispersion at (*β_2a_, p*s^2^) | -6.1×10^-6^ | Propagation loss (*α*, m^-1^) | 2.3 |

To correlate the electric field presented in the nonlinear Schrödinger equation (NLSE) and optical power utilized in the DREs, we apply boundary conditions in terms of the following protocols

$$\begin{aligned} P_{u}^{+}\left( 0,\lambda_{k} \right)=\frac{f\left| \left. \mathcal{F}\left[ u\left( 0,t \right) \right] \right|_{\omega=\omega_{k}} \right|^{2}\int\left| u\left( 0,t \right) \right|^{2}dt}{\sum_{i=1} \left| \left. \mathcal{F}\left[ u\left( 0,t \right) \right] \right|_{\omega=\omega_{i}} \right|^{2}} \end{aligned}$$

$$\begin{aligned} P_{v}^{+}\left( 0,\lambda_{k} \right)=\frac{f\left| \left. \mathcal{F}\left[ v\left( 0,t \right) \right] \right|_{\omega=\omega_{k}} \right|^{2}\int\left| v\left( 0,t \right) \right|^{2}dt}{\sum_{i=1} \left| \left. \mathcal{F}\left[ v\left( 0,t \right) \right] \right|_{\omega=\omega_{i}} \right|^{2}} \#\left( S10a \right) \end{aligned}$$

$$\begin{aligned} P_{u}^{-}\left( L_{f},\lambda_{k} \right)=\frac{f\left| \left. \mathcal{F}\left[ u\left( L_{f}^{+},t \right) \right] \right|_{\omega=\omega_{k}} \right|^{2}\int\left| u\left( L_{f}^{+},t \right) \right|^{2}dt}{\sum_{i=1} \left| \left. \mathcal{F}\left[ u\left( L_{f}^{+},t \right) \right] \right|_{\omega=\omega_{i}} \right|^{2}} \end{aligned}$$

$$\begin{aligned} P_{v}^{-}\left( L_{f},\lambda_{k} \right)=\frac{f\left| \left. \mathcal{F}\left[ v\left( L_{f}^{+},t \right) \right] \right|_{\omega=\omega_{k}} \right|^{2}\int\left| v\left( L_{f}^{+},t \right) \right|^{2}dt}{\sum_{i=1} \left| \left. \mathcal{F}\left[ v\left( L_{f}^{+},t \right) \right] \right|_{\omega=\omega_{i}} \right|^{2}}\#\left( S10b \right) \end{aligned}$$

where $f$ is the fundamental repetition rate.

**Fig. S5 Numerical simulation with different wavelength spacings of the MMI-mediated spectral filter.** **a** Optical spectra of dichromatic pulses with different wavelength spacings, i.e., varying from 7 to 12 THz. **b** Typical temporal evolutions for asynchronous (top) and synchronous (bottom) pulses. **c** Operation regimes are defined by the wavelength spacing of the MMI-mediated spectral filter.

Using this numerical model, we are able to study the effect of $\omega_{offset}$ on the generation of dichromatic pulses by precisely scanning the wavelength spacing within the transmission curve of the MMI-mediated spectral filter. As shown in **Figs. S5a,b**, by reducing 2$\omega_{offset}$ from 12 to 7 THz, the asynchronous pulses become synchronized, in which case the synchronization can be dominated by the cavity-induced soliton trapping effect imparted by the SESAM^5^. As shown in **Fig. S5c**, the MLFL with GHz-level fundamental repetition rate can operate in two distinguishing regimes that are defined by the wavelength spacing, i.e., bound state and dual-comb state.

**Fig. S6 Characteristics of the dichromatic pulses at different pump powers. a** Pulse energies of blue and red solitons as a function of pump power. The blue and red circles indicate the pulses centered around 1056 nm and 1062 nm, respectively. Two regimes are identified, wherein a composite state made of soliton singlet and doublet exists. **b,c** Optical spectra at the pump powers of 100 mW (**b**) and 200 mW (**c**) in simulation (top) and experiment (bottom).

Specifically, in the dual-comb state for the $\omega_{offset}=5.3$ THz, the simulated energies for the dichromatic pulses at around 1056 nm and 1062 nm are 117.3 pJ and 107 pJ, respectively. In the computation, the launched pump power is set to 100 mW, with a 3-dB coupling loss from the DF. For more details, intracavity pulse energies at different pump powers are concluded in **Fig. S6a**. Since the trapping effect in action can be primarily ascribed to the saturable absorption in GHz-repetition-rate fiber laser^4^, the change of pulse energy hardly affects the synchronization of the dichromatic pulses, but can give rise to a composite state comprised of soliton singlet and doublet with increasing pump power (see **Figs. S6b,c**). This theoretical prediction is also verified in the experiment.

**5.2 Intracavity collision of the asynchronous dichromatic pulses**

**Fig. S7 Evolutions of the asynchronous dichromatic pulse trains. a,b** Simulated round-trip evolving at 1056 nm (**a**) and 1062 nm (**b**). **c,d** Experimental pulse trains of the asynchronous dichromatic pulses at 1056 nm (**c**) and 1062 nm (**d**).

To gain more insight into asynchronous dichromatic pulses, we explore the dynamics of intracavity collision with a wavelength spacing 2$\omega_{offset}$ = 10.6 THz (corresponding to 6.3 nm, similar to the experiment). **Figures S7a** and **S7b** illustrate the simulated evolutions of the asynchronous dichromatic pulse trains at the two wavelengths, respectively. Assuming that the bandwidth of the detection system is remarkably lower than the optical bandwidth of the ultrashort pulse (here > THz), the peak intensity of the detected pulse can, to some extent, linearly represent the pulse energy. Thus, for comparison, the asynchronous dichromatic pulse trains at the two wavelengths captured by a 20-GHz bandwidth oscilloscope are provided in **Figs. S7c**,**d**.

**Fig. S8 Intracavity collision of the asynchronous dichromatic pulses. a,b** Temporal (**a**) and spectral (**b**) evolutions of the asynchronous dichromatic pulses. **c,d** Spectrograms of well-separated (**c**) and colliding (**d**) pulses. **e,f** Real-time spectral evolutions at 1056 nm (**e**) and 1062 nm (**f**). **g** Energy variations of the asynchronous dichromatic pulses.

Due to the difference in the fundamental repetition rates of the asynchronous dichromatic pulse trains, the collision every 6.75 μs is expected^6-8^. In the simulation, the collision evolution of the asynchronous dichromatic pulses in temporal and spectral domains are shown in **Figs. S8a** and **S8b**, respectively. There exists an intriguing spectral signature during the collision that the spectral edges of the short wavelength (i.e., blue pulse) and long wavelength (i.e., red pulse), which respectively manifest as convex-like and concave-like traits in the evolution landscapes. For the blue pulse with weaker peak intensity, its long-wavelength edge is enhanced by interacting with the red pulse when the SESAM is subjected to increasing incident power. The excess energy, induced at the long-wavelength edge, can be shed from the red pulse. The spectrograms of the well-separated and colliding pulses are illustrated in **Figs. S8c** and **S8d**, respectively. The pulses before collision are interpreted in the time-frequency plane (**Fig. S8c**, at the roundtrip of ~600), as well as the pulse during collision (**Fig. S8d**, at the roundtrip of ~765), as indicated by the dashed lines of **Figs. S8a**,**b**. The spectral-temporal pulse shaping imparted by the SESAM is recognized, as highlighted by the white arrow of **Fig. S8d**. The snapshots of the optical spectrum during the collision are also displayed in **Fig. S9**.

**Fig. S9** **Spectral evolution during** **the collision.** Snapshots of optical spectra at 1056 nm (blue) and 1062 nm (red) during the collision.

In the experiment, we capture the dynamic evolutions of the asynchronous dichromatic pulses using the time-stretch dispersive Fourier transform (TS-DFT) technology^9,10^. As shown in **Fig. S10**, the asynchronous dichromatic pulses at the two wavelength components are separated by the FWDM and counterpropagating with a pair of CIRs, in between there exists a 4-km length of SMF with a total dispersion of 90.65 ps^2^. The temporal pulse waveforms imparted with the spectral information are detected by two 10-GHz bandwidth photodiodes and recorded by a multi-channel 20-GHz high-speed oscilloscope. The temporal and spectral resolutions are 50 ps and 0.23 nm, respectively.

**Fig. S10 Experiment setup of the** **real-time detection system.** PD, photodiode; OSC, oscilloscope.

The spectral evolutions of the asynchronous dichromatic pulses are provided in **Figs. S8e**,**f**. As can be observed, the experimental colliding dynamic is in good agreement with the numerical simulation. The corresponding energy variations are shown in **Fig. S8g**.

**Supplementary** **Note 6: Other information about GHz dual-wavelength MLFL**

**6.1** **Operation regimes with varying pump power**

The mode-locking process experiences three states with varying pump power, i.e., Q-switching mode locking (QSML), single-wavelength mode locking (SWML), and dual-wavelength mode locking (DWML). As shown in **Fig. S11a**, the lasing threshold is about 25 mW. By increasing the pump power up to 60 mW, the laser operates at the QSML state. When the pump power further increases to 85 mW, the SWML state is achieved. As the pump power exceeds 100 mW, the mode-locking state transits from the SWML to DWML. The optical spectra are recorded by an OSA with a resolution of 0.02 nm, and the spectral characteristics of the QSML, SWML, and DWML states are illustrated in **Figs. S11****b**, **S11c** and **S11d**, respectively. It is noticed that, with increasing pump power, successful mode locking at 1062 nm occurs earlier than that at 1056 nm.

**Fig. S11 Operation regimes with varying pump power. a** Output power as a function of launched pump power. QSML, Q-switched mode locking; SWML, single-wavelength mode locking; DWML, dual-wavelength mode locking. **b-d** Optical spectra of QSML (**b**), SWML (**c**) and DWML (**d**). The blue and red curves represent the region at the wavelengths of 1056 nm and 1062 nm, respectively.

**6.2 Autocorrelation traces of the asynchronous dichromatic pulses**

We measure the autocorrelation traces of the asynchronous dichromatic pulses. As shown in **Figs. S12a**,**b**, the pulse widths at 1056 nm and 1062 nm are close, i.e., 3.29 ps and 3.24 ps, respectively, assuming the sech^2^ pulse shape.

**Fig. S12 Autocorrelation traces of the asynchronous dichromatic pulses at the wavelength of 1056 nm (a) and 1062 nm (b)**. The dashed curves represent the sech^2^ fitting.

**Supplementary Note 7:** **Long-term stability and** **noise characteristics of dual-wavelength MLFL**

**7.1 Long-term stability**

For practical applications, the long-term stability is significantly important. We monitor the optical spectrum of the DWML state at the pump power of 110 mW, as shown in **Fig. S13a**. The output power has a relative standard deviation (RSD) fluctuation of 0.29%, as shown in **Fig. S13b**.

**Fig. S13 Long-term stability of dual-wavelength MLFL. a** Spectral stability. **b** Output power stability.

**7.2 Noise characteristics**

The noise performance of the MLFL is shown in **Fig. S14** (blue curves at 1056 nm, red curves at 1062 nm). To avoid interference from another wavelength component during the noise measurement, the offset frequency is set from 10 Hz to 100 kHz. As illustrated in **Fig. S14a**, the single sideband phase noise of the asynchronous dichromatic pulses are measured at their corresponding fundamental carrier frequencies. The timing jitter integrated from the offset frequency range are calculated to be 5.042 ps and 1.929 ps, respectively, as shown in **Fig. S14b**. The relative intensity noise (RIN) are measured over a frequency range of 10 Hz to 100 kHz, as shown in **Fig. S14c**. The corresponding integrated RIN are calculated to be 0.082% and 0.044%, respectively, as illustrated in **Fig. S14d**.

**Fig. S14 Noise characteristics of the dual-wavelength MLFL.** **a** Single sideband phase noise. **b** Integrated timing jitter. **c** Relative intensity noise (RIN). **d** Integrated RIN.

**Supplementary Note 8: Dual-comb generation**

**8.1 Experimental setup of coherently spectral broadening**

**Figure S15** illustrates the experimental setup of dual-comb generation. Firstly, asynchronous dichromatic pulse trains are spectrally separated by using an FWDM with a cut-off wavelength of 1059 nm. Subsequently, both the wavelength components are individually amplified by the Yb-doped fiber amplifiers (YDFAs) to the average power of ~40 mW. Then, the asynchronous dichromatic pulses individually undergo nonlinear spectral broadening by passing through photonic crystal fiber (PCF, ~110 m in length, nonlinear coefficient of ~34 W^-1^km^-1^). Eventually, the separated asynchronous dichromatic pulse trains with broadened optical spectra are combined again by an OC.

**Fig. S15** **Experimental setup of the dual-comb generation.** YDFA, Yb-doped fiber amplifier; PCF, photonic crystal fiber; DCS, dual-comb spectroscopy.

**8.2** **Time domain signal of the DCS**

**Figure S16a** shows the typical time domain signal of the dual-comb laser after passing a low-pass filter with a cut-off frequency of 550 MHz. It can be found that, within one period (i.e., 6.75 μs), there exist two features^11,12^. One is the temporal interferogram resulting from the asynchronous optical sampling (ASOPS), as displayed in **Fig. S16b**. The other is the collision between the asynchronous dichromatic pulses. It is noticed that the position of the collision signal relative to the interferogram depends on the group delay between the dichromatic pulses accumulated in the extracavity path. To avoid aliasing, the fiber length in one arm of the interferometer must be adjusted to well separate the collision-induced signal from the interferogram.

**Fig. S16 Time domain signal of the DCS.** **a** Time domain signal after a low-pass filter in a span of 20 μs. **b** Closeup of the temporal interferogram.

**8.3** **Dual-comb** **characteristics with excessive spectral broadening**

For good coherence of the dual-comb source, it is particularly important to manipulate the optical power launched into the PCF for spectral broadening. **Figure S17a** showcases the degraded case when excessive spectral broadening is applied. In this case, even though it is characterized by a larger spectral overlap range, it leads to the occurrence of multiple interference structures, as depicted in **Fig. S17b**. Accompanying this, it corresponds to a multi-peak modulation pattern in the Fourier-transformed radio frequency (RF) spectrum, as shown in **Figs. S17c**,**d**, which largely influences the DCS applications.

**Fig. S17 Dual-comb characteristics with excessive spectral broadening. a** Output optical spectrum. **b** Temporal interferogram. **c,d** Fourier-transformed radio frequency (RF) spectrum in a wider (**c**) and a narrower (**d**) span.

**8.4** **Improving the optical bandwidth**

In the experiment, due to insufficient spectral broadening of the two wavelength components (resulting in inadequate spectral overlap), the optical bandwidth of the DCS shown in **Fig. 4c** is limited to about 1 THz, deviating from the maximum achievable optical bandwidth (i.e., 4.04 THz). To improve the optical bandwidth, extra efforts are required:

1. **Narrowing the center wavelength spacing of the dual-wavelength mode-locked fiber laser**: By reducing the center wavelength spacing between the two spectral components in dual-wavelength mode-locking, a broader spectral overlap can be obtained for a given spectral broadening, resulting in an optical bandwidth that approaches the theoretical maximum value. According to the theoretical calculation of the MMI-mediated spectral filtering effect provided in **Supplementary Note 2**, the center wavelength spacing of the generated asynchronous dichromatic pulses can be reduced without modifying the fundamental repetition rate by shortening the FMGF and lengthening the SMF. However, it should also be emphasized that the center wavelength spacing should not be excessively reduced. Otherwise, the insufficient group velocity difference provided by the small net dispersion can lead to the cavity-induced soliton trapping effect, causing the dichromatic pulses to bind together as a synchronous unit^5^ and thereby preventing the generation of asynchronous pulses (**Supplementary Note 5.1**).
2. **Expanding the spectral bandwidth of the dual-wavelength mode-locked fiber laser:** By performing dispersion management in the ultrashort cavity, such as utilizing a mirror with dichromatic functionality and high-dispersion characteristic as a cavity reflector^13-15^, or optimizing the parameters of SESAM^16^, a broader spectral bandwidth can be achieved. By applying these schemes, it can enable a larger spectral overlap at the same level of spectral broadening, thereby yielding a broader optical bandwidth of DCS.
3. **Optimizing the external spectral broadening:** We have already tried to achieve greater spectral overlap by increasing the amplification power to enhance the degree of spectral broadening, as detailed in **Supplementary Note 8.3**. While this approach is effective in enhancing the optical bandwidth of DCS (**Fig. S17c**), the excessive accumulation of nonlinear effects compromises the coherence, thereby substantially limiting the DCS applications. In future work, the nonlinear fiber amplification technology can potentially be applied to optimize spectral broadening^17^, achieving a high-quality spectral overlap while preserving the coherence property.

**Supplementary Note 9: Comparison of single-cavity dual-comb sources**

**Table** **S2** summarizes the relevant reports on single-cavity dual-comb sources based on dual-wavelength mode-locked fiber lasers and outlines their key performance characteristics. For intuitive comparison, the refresh time ($\Delta T=1/\Delta f$, where $\Delta f$ is frequency rate difference), and maximum optical bandwidth ($\upsilon={f^{2}}/\left( 2\Delta f \right)$) of the DCS are plotted on the x- and y-axis, respectively, as shown in **Fig. S18**. It is obvious that, the single-cavity dual-comb source in our work achieves an acquisition speed several orders of magnitude faster than that of previous work, and the maximum achievable optical bandwidth is still maintained at an even higher level. This benefits from the high $\Delta f$ and $f$ in the dual-wavelength mode-locked fiber laser.

**Table S2** Performances of single-cavity dual-comb sources.

| **Fundamental repetition rate (MHz)** | **Repetition rate difference (kHz)** | **Refresh time (ms)** | **Maximum optical bandwidth (THz)** | **Reference** |
| --- | --- | --- | --- | --- |
| 77 | 2.6 | 0.38 | 1.14 | [11] |
| 71.88 | 3.27 | 0.31 | 0.79 | [12] |
| 52.74 | 1.25 | 0.8 | 1.11 | [18] |
| 58.69 | 1 | 1 | 1.72 | [19] |
| 24.83 | 0.633 | 1.58 | 0.49 | [20] |
| 42.4 | 0.86 | 1.16 | 1.05 | [21] |
| 50.56 | 2 | 0.5 | 0.64 | [22] |
| 92.45 | 1.95 | 0.51 | 2.19 | [23] |
| 10.42 | 1.05 | 0.95 | 0.05 | [24] |
| 154.6 | 3.635 | 0.28 | 3.29 | [25] |
| **1093** | **148** | **0.0068** | **4.04** | **This work** |

**Fig. S18** **Refresh time and maximum optical bandwidth of single-cavity dual-comb sources based on dual-wavelength mode-locked fiber lasers.**

**Supplementary Note 10:** **Reflection wavelength variation of the fiber Bragg grating (FBG) subjected to strain**

**10.1** **Optical spectra measured by the DCS and OSA**

As shown in **Fig. S19**, the reflected optical spectra of FBGs are successfully reconstructed by applying the Fourier transform to the temporal interferograms. With varying voltages applied to the piezoelectric (PZT), i.e., 0 V, 50 V and 100 V, the optical spectra measured by both the DCS and standard OSA are consistent, demonstrating the precise spectral measurement capability of the GHz single-cavity all-fiber dual-comb system.

**Fig. S19** **Reflected optical spectra of fiber Bragg gratings (FBGs).** Reflected optical spectra of FBGs measured by the DCS (green) and OSA (black) with different voltages applied to the PZT, i.e., 0 V, 50 V and 100 V, respectively.

**10.2 Relationship between the change of the reflection** **wavelength and the strain applied on the FBG**

According to the specification of the PZT, a 100-V voltage change can result in a 20-μm displacement, i.e., a 20-μm deformation in the grating area of the FBG with a 1-cm length, corresponding to a 2-mε strain. As discussed in **Fig. 5b**, a 100-V voltage can change the reflection wavelength of the FBG2 by 311 pm, leading to a relationship between wavelength variation and applied strain is 1 nm = 6.41 mε.

**Supplementary Note 11:** **Fabrication of the FBG-based probe**

To secure the FBG from the damage of the strong shock generated upon explosion, we utilize polydimethylsiloxane (PDMS), a thermoset elastomer, to constitute the FBG probe. Firstly, the PDMS precursor is prepared by mixing the monomer and curing agent with a weight ratio of 10:1, followed by carefully removing air bubbles. As shown in **Fig. S20a**, the PDMS is injected into a homemade 3D-printed mold (2 cm × 1 cm size with 200 μm thickness) through a syringe and covered with the straightened FBG placed inside the mold. After being heated at 80 ℃ for about 100 minutes, the FBG-based probe is successfully fabricated, as shown in **Fig. S20b**.

**Fig. S20 Fabrication of the FBG-based probe. a** Fixing the FBG2 with polydimethylsiloxane (PDMS). **b** Photograph of the FBG-based probe.

**Supplementary Reference**

1. Jiang, Z. & Marciante, J. R., Impact of transverse spatial-hole burning on beam quality in large-mode-area Yb-doped fibers. *J. Opt. Soc. Am. B* **25**, 247–254 (2008).
2. Manuylovich, E. S., Dvoyrin, V. V. & Turitsyn, S. K. Fast mode decomposition in few-mode fibers. *Nat. Commun.* **11**, 5507 (2020).
3. Cheng, H., Lin, W., Zhang, Y., Jiang, M., & Luo, W. Numerical insights into the pulse instability in a GHz repetition-rate thulium-doped fiber laser. *J. Lightwave Technol.* **39**, 1464–1470 (2021).
4. Wang, W. et al. Gain-guided soliton: Scaling repetition rate of passively modelocked Yb-doped fiber lasers to 12.5 GHz. *Opt. Express* **27**, 10438–10448 (2019).
5. Lin, W. et al. Vector soliton dynamics in a high-repetition-rate fiber laser. *Opt. Express* **29**, 12049–12065 (2021).
6. Cui, Y. et al. Dichromatic “breather molecules” in a mode-locked fiber laser. *Phys. Rev. Lett.* **130**, 153801 (2023).
7. Liu, M., Li, T. J., Luo, A. P., Xu, W. C., & Luo, Z. C. “Periodic” soliton explosions in a dual-wavelength mode-locked Yb-doped fiber laser. *Photon. Res.* **8**, 246–251 (2020).
8. Wei, Y., Li, B., Wei, X., Yu, Y. & Wong, K. K. Y. Ultrafast spectral dynamics of dual-color-soliton intracavity collision in a mode-locked fiber laser. *Appl. Phys. Lett.* **112**, 081104 (2018).
9. Goda, K. & Jalali, B. Dispersive Fourier transformation for fast continuous single-shot measurements. *Nature Photon.* **7**, 102–112 (2013).
10. Tong, Y. C., Chan, L. Y. & Tsang, H. K. Fiber dispersion or pulse spectrum measurement using a sampling oscilloscope. *Electron. Lett.* **33**, 983–985 (1997).
11. Fellinger, J. et al. Tunable dual-comb from an all-polarization-maintaining single-cavity dual-color Yb: fiber laser. *Opt. Express* **27**, 28062–28074 (2019).
12. Liao, R. et al. Dual-comb spectroscopy with a single free-running thulium-doped fiber laser. *Opt. Express* **26**, 11046–11054 (2018).
13. Cheng, H., Chen, K., Tao, Y. & Shao, W. Dissipative Solitons in Centimeter-Scale Fiber Lasers. *J. Lightwave Technology* **41**, 6779–6785 (2023).
14. Zhang, Y., et al. Multifunctional highly dispersive mirror for fiber oscillator. *Opt. Express* **29**, 43174–43181 (2021).
15. Chen, H., Chang, G., Xu, S., Yang, Z. & Kärtner, F. 3 GHz, fundamentally mode-locked, femtosecond Yb-fiber laser. *Opt. Lett.* **37**, 3522–3524 (2012).
16. Song, D., et al. Theoretical and experimental investigations of dispersion-managed, polarization-maintaining 1-GHz mode-locked fiber lasers. *Opt. Express* **31**, 1916–1930 (2023).
17. Wang, W., et al. High-speed wavelength-swept femtosecond source from 1055 to 1300 nm using a GHz femtosecond fiber laser. *Opt. Lett.* **47**, 1677–1680 (2022).
18. Zhao, X. et al. Picometer-resolution dual-comb spectroscopy with a free-running fiber laser. *Opt. Express* **24**, 21833–21845 (2016).
19. Lin, B. et al. Dual-comb absolute distance measurement based on a dual-wavelength passively mode-locked laser. *IEEE Photonics J.* **9**, 7106508 (2017).
20. Zhao, K. et al. Free-running dual-comb fiber laser mode-locked by nonlinear multimode interference. *Opt. Lett.* **44**, 4323–4326 (2019).
21. Gu, X. et al. Ultrashort pulse duration and broadband dual-comb laser system based on a free-running passively mode-locked Er-fiber oscillator. *Laser Physics Lett.* **18**, 125101 (2021).
22. Hu, D. et al. Dual-comb absolute distance measurement of non-cooperative targets with a single free-running mode-locked fiber laser. *Opt. Commun.* **482**, 126566 (2021).
23. Fellinger, J. et al. Simple approach for extending the ambiguity-free range of dual-comb ranging. *Opt. Lett.* **46**, 3677–3680 (2021).
24. Pu, G. et al. Intelligent single-cavity dual-comb source with fast locking. *J. Lightwave Technology* **41**, 593–598 (2023).
25. Li, Y., Zhang, J., Wu, F., Liu, G. & Xiao, X. Fast dual-comb spectroscopy based on a dual-wavelength all-fiber ring laser with high repetition rate. *Applied Physics Express* **16**, 012015 (2023).
